# Anterior gradient-2 plays a critical role in breast cancer cell growth and survival by modulating cyclin D1, estrogen receptor-α and survivin

**DOI:** 10.1186/bcr2586

**Published:** 2010-06-04

**Authors:** Kathryn E Vanderlaag, Susan Hudak, Laura Bald, Laurence Fayadat-Dilman, Manjiri Sathe, Jeff Grein, Mary J Janatpour

**Affiliations:** 1 Schering-Plough Biopharma, 901 South California Avenue, Palo Alto, CA 94304, USA; 2 Novartis Institutes for BioMedical Research, 4560 Horton Street M/S 4.4, Emeryville, CA 94608, USA

## Abstract

**Introduction:**

Anterior-gradient 2 (AGR2) is an estrogen-responsive secreted protein. Its upregulation has been well documented in a number of cancers, particularly breast cancer, for which mixed data exist on the prognostic implications of AGR2 expression. Although emerging evidence indicates that AGR2 is associated with poor prognosis, its function and impact on cancer-relevant pathways have not been elucidated in breast cancer.

**Methods:**

To investigate the biologic role of AGR2 in breast cancer, AGR2 was transiently knocked down, by using siRNA, in T47 D and ZR-75-1 (estrogen receptor-α (ER)-positive) and MDA-MB-231 and SK-BR-3 (ER-negative) human breast cancer cell lines. The impact of silencing AGR2 was evaluated in both anchorage-dependent and anchorage-independent growth (soft agar, spheroid) assays. Cell-cycle profiles in ER-positive cell lines were determined with BrdU incorporation, and cell death was measured with Annexin V, JC-1, and F7-26 staining. After transiently silencing AGR2 or stimulating with recombinant AGR2, modulation of key regulators of growth and survival pathways was assessed with Western blot. Combination studies of AGR2 knockdown with the antiestrogens tamoxifen and fulvestrant were carried out and assessed at the level of anchorage-dependent growth inhibition and target modulation (cyclin D1, ER).

**Results:**

AGR2 knockdown inhibited growth in anchorage-dependent and anchorage-independent assays, with a more-pronounced effect in ER-positive cell lines. Cyclin D1 levels and BrdU incorporation were reduced with AGR2 knockdown. Conversely, cyclin D1 was induced with recombinant AGR2. AGR2 knockdown induced cell death in ZR-75-1 and T47 D cells, and also downregulated survivin and c-*Myc*. Evidence of AGR2-ER crosstalk was demonstrated by a reduction of ER at the protein level after transiently silencing AGR2. AGR2 knockdown in combination with fulvestrant or tamoxifen did not preclude the efficacy of the antiestrogens, but enhanced it. In addition, p-Src, implicated in tamoxifen resistance, was downregulated with AGR2 knockdown.

**Conclusions:**

Transiently silencing AGR2 in ER-positive breast cancer cell lines inhibited cell growth and cell-cycle progression and induced cell death. Breast cancer drivers (ER and cyclin D1) as well as cancer-signaling nodes (pSrc, c-*Myc*, and survivin) were demonstrated to be downstream of AGR2. Collectively, the data presented support the utility of anti-AGR2 therapy in ER-positive breast cancers because of its impact on cancer-relevant pathways.

## Introduction

In the United States, one in eight women will be diagnosed with breast cancer in their lifetimes, and the incidence is increasing worldwide [1]. Estrogen receptor-α (ER)-positive breast cancer accounted for 75% of breast cancer cases in the United States between 1992 and 1998 [2]. 17β-Estradiol (E2) is the ligand for ER and exerts its action by upregulating a number of key mediators, including cyclin D1 and c-*Myc *[3,4]. Cyclin D1 is overexpressed in >50% and amplified in 15% of breast cancer cases [5] and acts as a mitogenic sensor [6] by responding to oncogenes and various growth factors, including E2. It plays a critical role in cell-cycle progression, as evidenced by the lack of entry into S phase in the absence of cyclin D1 [7]. In addition, cyclin D1-deficient mice do not grow breast tumors when induced by the oncogenes *Ras *and *Neu *[8], further supporting cyclin D1 as a key driver in certain breast tumors.

Because of its impact on cell-growth and -survival pathways, E2 signaling has proven to be an efficacious target for ER-positive breast cancer therapy. However, approximately half of ER-positive tumors have an intrinsic resistance to endocrine therapy, and 30% to 40% of the remaining responsive population will acquire resistance to tamoxifen [9], thus necessitating the exploration of alternative therapeutic targets.

Anterior gradient-2 (AGR2) is a secreted protein that was originally identified to be coexpressed with ER in breast cancer cell lines [10]. AGR2 has since been demonstrated to be estrogen [11-13] and androgen responsive [14], and its upregulation has been reported in a number of cancers, including breast, lung, ovarian, gastric, pancreatic, esophageal, and prostate cancer [11,15-25]. Additionally, in the ER-negative breast cancer cell line, MDA-MB-231, AGR2 was induced under serum starvation and hypoxia [26], suggesting a role for AGR2 in physiologically relevant stress conditions. Early expression studies have correlated AGR2 expression with a better prognosis [18], possibly because of its positive association with ER-positive tumors, which typically have a more-favorable prognosis than do their ER-negative counterparts [27]. Subsequent studies have explored the ER-positive tumor population and shown that AGR2 is inversely associated with overall and relapse-free survival [21,25], prompting us to ask whether AGR2 plays a critical role in more-invasive ER-positive tumors.

Literature relating to the functional role of AGR2 in cancer is limited in scope. In the premalignant Barrett esophagus and esophageal cancer models, AGR2 overexpression induces colony formation and transformation [15,28]. In the course of this investigation, the converse, siRNA- or shRNA-mediated AGR2 knockdown, was shown to inhibit colony and subcutaneous growth in esophageal and pancreatic cancer models [24,28]. In breast cancer models, overexpression of AGR2 failed to alter tumor formation *in vivo *or growth rate *in vitro*, but, rather, reduced cell adhesion and increased the numbers of metastases [11]. Although the phenotypic observations in these articles are compelling, very little signaling downstream of AGR2 has been elucidated.

AGR2 warranted further evaluation of its biology based collectively on its prevalence in breast cancer, its negative correlation with patient survival within the ER-positive breast cancer subpopulation, and literature implications of a functional role in cancer. To evaluate the impact of targeting AGR2 in cancer, siRNA was used to knock down AGR2 in breast cancer cell lines that endogenously express AGR2 at varying levels. Phenotypic effects on cell proliferation and death, as well as modulation of key cancer-signaling nodes, including cyclin D1, c-*Myc*, p-*Src*, and survivin, were observed. These pathways were conversely modulated on treatment of a breast cancer line with recombinant AGR2. Combining AGR2 knockdown with ER antagonists resulted in enhanced antiproliferative effects on ER-positive lines. Altogether, our results demonstrate a critical role for AGR2 in breast cancer growth and survival, identify downstream signaling of AGR2, and thus support AGR2 as a promising oncology target for therapeutic agents.

## Materials and methods

### Cell lines and chemicals

Human breast cancer cell lines T47 D, ZR-75-1, MCF-7, MDA-MB-231, and SKBR3 were purchased from ATCC. Cell lines were maintained per ATCC guidelines. The cells were grown at 37°C in a humidified atmosphere with 5% CO_2_. The 17β-estradiol, ICI 182,780, MG132, 4-hydroxytamoxifen were obtained from Sigma. Propidium iodide/RNase solution was purchased from BD Biosciences.

### Detection of AGR2 in supernatant

Conditioned media from cell lines was collected and centrifuged to remove cell debris. AGR2 was immunoprecipitated (IP) from 2-mL samples with Protein A/G beads, 40 μg mouse anti-AGR2 (Novus) or isotype control, and overnight incubation. IP samples were analyzed with Western blot (described later).

#### siRNA knockdown

**Figure 1:**
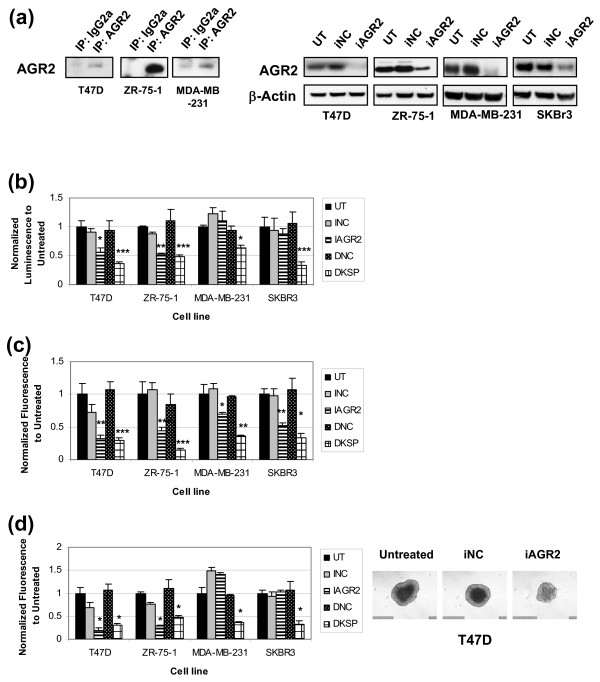
**siRNA-mediated AGR2 knockdown affects anchorage-dependent and anchorage-independent growth in breast cancer cell lines**. T47 D, ZR-75-1, MDA-MB-231, and SK-BR-3 cells were transfected with negative control siRNA (iNC), AGR2 siRNA (iAGR2), or untransfected (UT). KSP (DKSP) and its corresponding control (DNC) were used as transfection controls. Results are expressed as a ratio of untransfected cells (±SD), n = 3. **(a) **Detection of endogenous AGR2 in breast cancer cell line supernatants by IP-Western and whole-cell lysates by Western. AGR2 knockdown was confirmed in lysates 72 hours after transfection. β-Actin served as a loading control. **(b) **The impact of iAGR2 on anchorage-dependent growth was evaluated at 96 hours after transfection by using the Cell Titer Glo assay. Anchorage-independent growth assays were also used: (i) soft agar colony formation assay **(c)**, with Alamar blue as a readout; (ii) spheroid assay **(d)**, in which lysed spheroid LDH levels were representative of total cell number after 8 days; corresponding spheroid images were also captured. *P < 0.05; **P < 0.01; ***P < 0.001.

**Figure 2:**
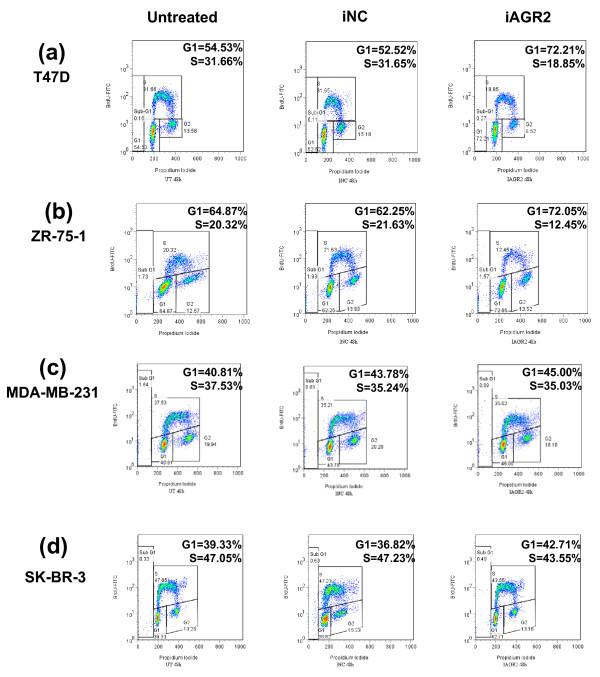
**AGR2 knockdown reduces cell proliferation in ER-positive breast cancer cells**. Cell-cycle profiles were analyzed with BrdU incorporation. Cells were pulse-labeled with 10 μM BrdU 48 hours after transfection and analyzed for BrdU incorporation with FACS. Cells were gated on Sub G_1_, G_0_/G_1_, S, and G_2_/M populations. **(a) **T47 D, **(b) **ZR-75-1, **(c) **MDA-MB-231, and **(d) **SK-BR-3 cells.

**Figure 3:**
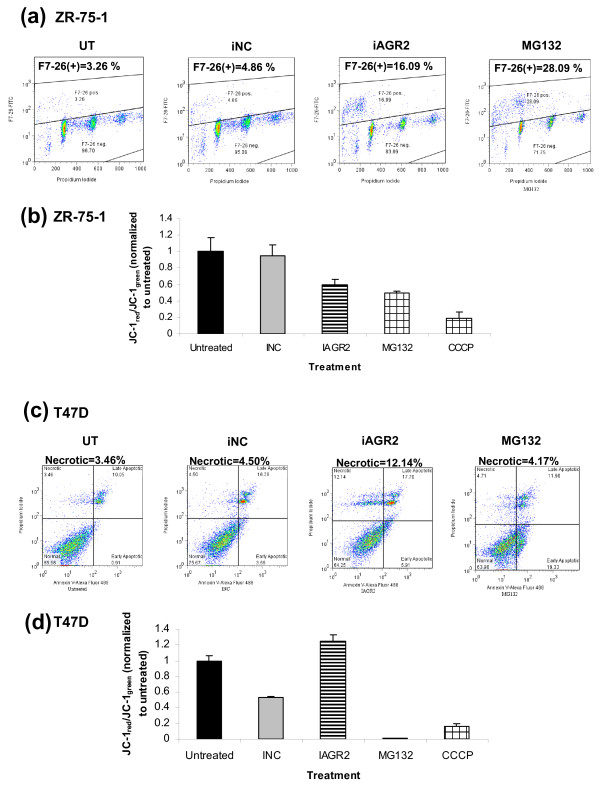
**AGR2 knockdown induces cell death**. ZR-75-1 cells were collected 96 hours after AGR2 knockdown and analyzed for cell death by measuring ssDNA breaks by using the following: **(a) **F7-26 staining by FACS analysis, and **(b) **alterations in mitochondrial membrane potential by determining the ratio of JC-1_red _to JC-1_green _and represented as a ratio of the untransfected control (±SD), n = 3. MG132 and CCCP served as apoptosis and depolarization controls, respectively. Cell death was investigated 96 hours after AGR2 knockdown in T47 D cells by using the following: **(c) **annexin V (AV) and propidium iodide (PI) and gated on normal (AV^-^/PI^-^), necrotic (AV^-^/PI^+^), early apoptotic (AV^+^/PI^-^), and late apoptotic/necrotic (AV^+^/PI^+^) cells; and **(d) **JC-1 staining, as previously described.

**Figure 4:**
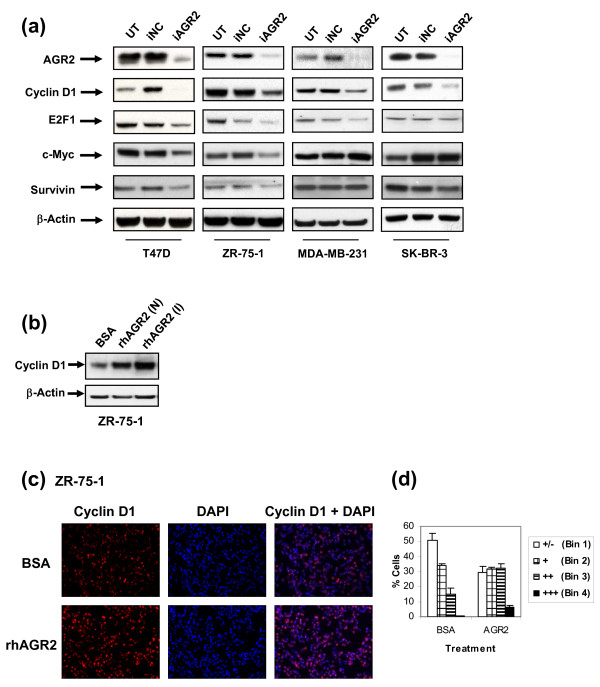
**Target modulation of proliferation and survival proteins by AGR2**. **(a) **Lysates 72 hours after transfection with nontargeting control (iNC) or AGR2 (iAGR2) were evaluated with Western blot for modulation of regulators of growth and survival. Note: Some blots may be from different gels run with the same set of samples. **(b) **ZR-75-1 cells were treated for 6 hours with BSA (5 μg/mL), Novus rhAGR2 (rhAGR2 (N)), or in-house rhAGR2 (rhAGR2(I)), and analyzed with Western blot for cyclin D1 induction. **(c) **ZR-75-1 cells were plated in eight-well chamber slides and treated with 5 μg/mL of BSA or rhAGR2 (I) for 6 hours. Cells were stained with cyclin D1, and mounting medium containing DAPI was used. Images were taken by using a fluorescent microscope and pseudo-colored in Adobe Photoshop. **(d) **Quantitation of cyclin D1 immunofluorescence images. The percentage of cells in each bin based on cyclin D1 intensity is represented (Bin 1, weakest staining; Bin 4, brightest staining). Results are expressed as the mean ± SD; n = 4.

**Figure 5:**
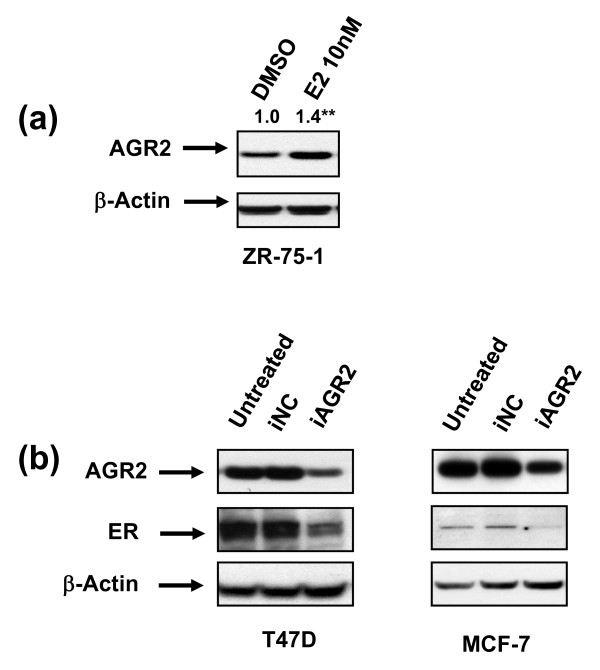
**Evidence of ER-AGR2 crosstalk**. **(a) **ZR-75-1 cells were treated with vehicle control (DMSO) or E2 (10 n*M*) for 24 hours analyzed with Western blot. Numbers above bands represent relative AGR2 induction with E2 treatment after quantitation and normalized to β-actin (Image J). **(b) **Lysates 72 hours after AGR2 knockdown were analyzed with Western blot for ER. **P < 0.01. Note: Some blots may be from different gels run with the same set of samples.

**Figure 6:**
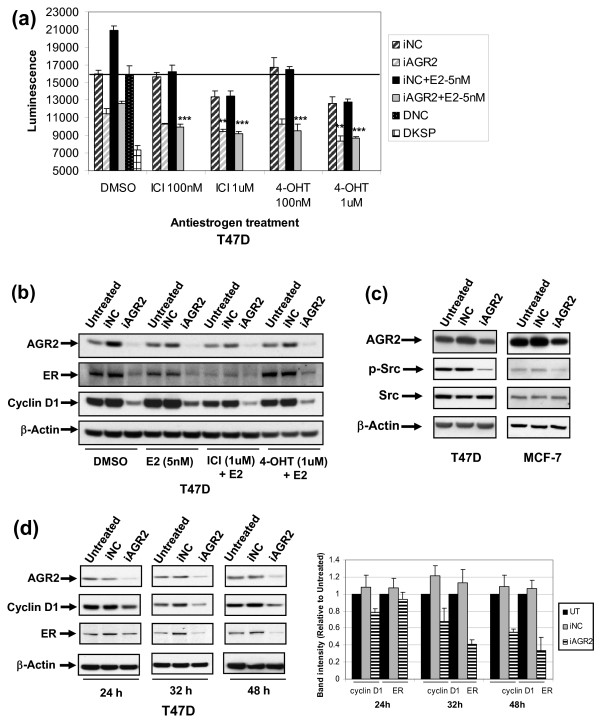
**ER-independent activities after AGR2 knockdown**. T47 D cells were transfected with iNC or iAGR2, and after 24 hours, cells were treated in combination with 4-hydroxytamoxifen (4-OHT) or ICI 182,780 (ICI) at doses of 100 n*M *or 1 μ*M*. The growth-inhibitory effects of combination treatments were assessed with Cell Titer Glo 96 hours after transfection **(a)**, or the level of target modulation at 72 hours with Western blot analysis **(b)**. **(c) **Modulation of p-Src was analyzed 72 hours after AGR2 knockdown. The kinetics of cyclin D1 and ER modulation were determined with Western blot, and densitometric values of cyclin D1 and ER were calculated and normalized to β-actin (Image J) and represented as the mean ± SD, n = 3. *P < 0.05; **P < 0.01; ***P < 0.001, antiestrogen and AGR2 knockdown combination versus AGR2 knockdown alone. Note: Some blots may be from different gels run with the same set of samples.

Cells were transfected in Optimem (Gibco) containing 100 nmol/L iRNA pools by using Dharmafect 3 (Dharmacon) for SK-BR-3 cells or Dharmafect 4 for all other cell lines for 24 hours, according to the manufacturer's protocol. Invitrogen AGR2 pooled siRNA, Invitrogen stealth negative control, KSP positive control (Dharmacon), or Dharmacon negative control siRNA was used. To support the effects observed after siRNA knockdown in Figures 1, 2, 3, 4, 5 and 6 as being AGR2-specific effects and not an off-target effect of the Invitrogen siRNA reagent, additional AGR2 siRNA reagents were used. siRNA reagents targeting distinct sequences from Ambion (Ambion Silencer) and Dharmacon (On-Target Plus Smartpool) were used in anchorage-dependent functional studies in T47 D and MDA-MB-231 cells with effects similar to those seen with the Invitrogen reagent (see Supplementary figure S1a in Additional file 1).

#### Cell Titer Glo viability assay

Twenty-four hours after transfection, 5 × 10^3 ^cells were plated in black-view plates. Cell Titer Glo reagent (Promega) was added according to the manufacturer's directions 96 h after transfection. The luminescence was read with a multiwell plate reader (DTX 880) and an integration time of 100,000 μsec.

#### Soft agar colony formation assay

96-well flat-bottom plates were coated with 1% agarose. Twenty-four hours after transfection, 5 × 10^2 ^cells/well were added in media with a final concentration of 0.33% agarose. After a 7-day incubation, Alamar blue (Biosource) was added to each well, and the fluorescence was read by using a multiwell plate reader at 530/590 nm.

#### Spheroid assay

Twenty-four hours after transfection, cells were plated at a density of 1 × 10^4 ^cells/well in 96-well round-bottom plates coated with Polyhema (Sigma) and grown on a waver platform. The spheroids were lysed in a final concentration of 2% Triton X-100 after 7 days. The LDH kit (Promega) was used according to the manufacturer's directions for lysed spheroids and read on a plate reader at 560/590 nm.

#### Cell-cycle analysis by BrdU incorporation

Cell-cycle analysis was determined by transfecting cells with siRNA, harvesting (adherent and suspension) cells after 48 h, and measuring BrdU incorporation, as previously described [29]. Samples were then analyzed by using a FACSCanto (Becton Dickinson), and FlowJo was used to quantitate the cell-cycle distribution.

#### Detection of cell death by Annexin V/Propidium Iodide staining

Cells were plated at a density of 1 × 10^5 ^cells/mL and treated with AGR2 siRNA for 96 hours. Floating and adherent cells were stained with Annexin V-Alexa Fluor 488 (Invitrogen), as described in the manufacturer's protocol, and resuspended in PI/RNase. Samples were run within 30 minutes with flow cytometry, and plots were analyzed in FlowJo.

#### Detection of ssDNA breaks by F7-26 staining

Detection of apoptosis was determined 96 hours after AGR2 siRNA treatment by collection of floating and adherent cells and fixation in 100% methanol overnight. Cells were then stained for F7-26, as previously described [30]. Cells were resuspended in PI/RNase solution and analyzed by using a FACSCanto. FACS plots were analyzed in FlowJo.

#### Measurement of mitochondrial membrane potential by JC-1

Cells were plated at a density of 1 × 10^5 ^cells/six-well plate and were harvested 96 hours after AGR2 siRNA treatment. Cells were stained with JC-1 (Invitrogen) according to the manufacturer's directions, including the positive-control CCCP. Samples were analyzed by using a FACSCanto, and CCCP-treated samples were used to perform standard compensation. The ratio of JC-1_aggregate_/JC-1_monomer _was determined by calculating the geometric mean of PE/FITC in FlowJo and then expressed as a ratio of the untransfected control population.

#### Western blot analysis

Lysates were isolated by using the RIPA buffer (Sigma) containing phosphatase and protease inhibitors, according to the manufacturer's directions. Western blot analyses were done in 2% to 5% milk/0.05% TBS-Tween at a dilution of 1:1,000 overnight at 4°C. Antibodies were purchased from the following vendors: Novus (AGR2), Santa Cruz Biotechnology (ER), Epitomics (cyclin D1, c-*Myc*), Cell Signaling (cyclin D1, E2F1, p-Erk, Erk, p-Src, Src, survivin). Secondary conjugated sheep anti-mouse IgG (Amersham) and donkey anti-rabbit IgG (Amersham) HRP antibodies were used at 1:5,000 for an hour. Blots were developed by using chemiluminescent reagents (Pierce). Densitometry was performed on cyclin D1, ER, and β-actin bands by using ImagePro (NIH), and a ratio of cyclin D1 or ER values to loading control (β-actin) values for each treatment was computed. Ratios were expressed relative to untransfected samples from the same time point.

#### Treatment of ZR-75-1 cells with E2

ZR-75-1 cells were plated at a density of 1.25 × 10^5 ^cells/mL in RPMI containing 2% charcoal-stripped serum. Twenty-four hours after plating, the medium was removed, and E2 was then added at a final concentration of 10 n*M*, and protein was isolated after 24 hours, as described previously.

#### Cyclin D1 detection in ZR-75-1 cells treated with rhAGR2

ZR-75-1 cells were plated in six-well plates or Lab-Tek II chamber slides (Nalge Nunc) in reduced serum and treated with 5 μg/mL of BSA, rhAGR2 (in-house), or full-length rhAGR2 (Novus). Protein from six-well plates was isolated 6 hours after treatment, as described previously. Slides were fixed 6 hours after rhAGR2 treatment with 10% neutral buffered formalin, blocked in 3% BSA + 0.5% Triton × in PBS, incubated overnight with anti-cyclin D1 Ab (Epitomics), 1:200, and for 1 hour with Alexa 594-goat anti-rabbit (Invitrogen), 1:500. Slides were mounted by using Pro-Long Gold with DAPI, and the slides were imaged with a fluorescent microscope (Leica). Quantitation of cyclin D1 intensity was performed by using ImagePro by generating a DAPI-based nuclear map and determining the relative cyclin D1 intensity per nucleus, which was subsequently separated into four bins based on relative intensity. In-house rhAGR2 was purified from mammalian HEK293E cells overexpressing N-terminal His-tagged recombinant AGR2.

#### E2 and/or antiestrogen treatment

T47 D cells were plated at a density of 1 × 10^5 ^cells/mL in 10% charcoal-stripped RPMI and transfected, as previously described. At 24 hours after transfection, the medium was changed to 5% charcoal-stripped RPMI, and cells for functional assays were replated at a density of 3 × 10^3 ^cells/well for Cell Titer Glo and treated with vehicle, E2, or antiestrogens. Protein was isolated 72 hours after transfection, and phenotype plates were assayed 96 to 120 hours after transfection.

#### Generation of monoclonal rat anti-AGR2 Ab and test of AGR2 Ab specificity and cross-reactivity by ELISA

One Lewis rat was immunized with human recombinant AGR2 generated in-house for 4 months. The inguinal and popliteal nodes were subsequently harvested and fused with electrofusion by using Cyto PulseSciences Model PA-101C Electrofusion System. To test for specificity and cross-reactivity, mouse AGR2, human AGR2, and AGR3 ELISAs were used. Plates were coated with in-house human AGR2, human AGR3, or mouse AGR2 at 0.5 μg/mL (50 μL total volume in PBS) overnight at 4°C. Supernatant from fusions (50 μL) were incubated on coated plates for 1 hour at room temperature, followed by 1:2,000 of goat anti-rat HRP Ab (Jackson Laboratories) for 30 minutes at room temperature. ABTS (50 μL/well) was added to wells and incubated for 10 minutes at room temperature. Absorbance was read at 405 nm.

#### Anti-AGR2 Ab treatment of T47 D cells

Cells were plated in 96-well flat-bottom plates at 1,000 cells/well for growth assays or Lab-Tek II chamber slides in low (2%) serum for cyclin D1 immunofluorescence studies. Cells plated in chamber slides were treated with the rat anti-AGR2 Ab generated in-house twice in a 48-hour period at a concentration of 10 μg/mL and stained for cyclin D1, as previously described. For growth studies, 48 hours after plating, antibodies were added to 96-well plates at a final concentration of 20 μg/ml. After an additional 5 days of incubation, MTT Reagent (Roche) was added according to the manufacturer's instructions. After 4 hours, Lysis Buffer was added, and plates were incubated an additional 24 hours. Plates were read by using a multiwell plate reader at 550/690 nm.

### Statistical analysis

A two-tailed Student *t *test was used for statistical analysis of comparative data by using GraphPad Prism. Data were expressed as means of at least three independent experiments ± SD, with *P *< 0.05 considered statistically significant.

## Results

### AGR2 knockdown in breast cancer cells affects anchorage-dependent and anchorage-independent growth

Previous reports have shown that knocking down AGR2 has an impact on growth in esophageal and pancreatic cancer cell lines [24,28]. The role of endogenous AGR2 in breast cancer cell proliferation was assessed with anchorage-dependent and anchorage-independent growth assays. Four breast cancer cell lines, two ER-positive (T47 D and ZR-75-1) and two ER-negative (MDA-MB-231, SK-BR-3), were used. All cell lines were transiently transfected with siControl and siAGR2. Endogenous AGR2 expression was detected in cell-line supernatants and whole-cell lysates (Figure 1a), and knockdown of AGR2 protein (Figure 1a, right) was confirmed up to 96 hours after transfection (Supplementary figure S1b in Additional file 1). Silencing AGR2 inhibited anchorage-dependent proliferation and anchorage-independent spheroid growth only in ER-positive cell lines (Figure 1b, d). Similar results on anchorage-dependent growth in ER-positive T47 D and ER-negative MDA-MB-231 cells were obtained by using multiple AGR2 siRNA reagents that targeted distinct sequences (Supplementary figure S1a in Additional file 1). In all four cell lines, AGR2 knockdown inhibited anchorage-independent growth in the soft agar assay (Figure 1c). Collectively, these data demonstrate that AGR2 knockdown has a negative impact on anchorage-dependent and anchorage-independent growth that is more pronounced in ER-positive breast cancer cells.

### AGR2 knockdown has an impact on cell cycle and induces cell death

To determine whether the phenotype from Figure 1 was a result of growth inhibition or cell death or both, cell-cycle profiles and apoptosis/necrosis assays were used. In T47 D and ZR-75-1 cells, transient knockdown of AGR2 significantly reduced the percentage of cells in S phase >40% and increased the percentage of cells in G_0_/G_1 _phase compared with the transfection control (Figure 2a, b). By contrast, treatment with siAGR2 in the two ER-negative cell lines, MDA-MB-231 and SK-BR-3 cells, failed to alter the cell-cycle profile compared with control cells (Figure 2c, d).

Transient knockdown of AGR2 in ZR-75-1 cells yielded an 11% increase in F7-26 staining, indicative of single-stranded DNA breaks and apoptosis (Figure 3a). An apoptotic phenotype with AGR2 knockdown in ZR-75-1 cells was further supported by an observed depolarization of the mitochondrial membrane, established by using JC-1 staining, that was comparable to the apoptosis-inducing control, MG132 (Figure 3b). By contrast, in T47 D cells, AGR2 silencing increased the percentage of the propidium-iodide-only cell population >7% above both the negative control and the apoptosis control, which is indicative of necrosis. Although the increase in propidium iodide staining was moderate, we consistently observed this result (n = 3). The Annexin V-positive population was only slightly higher compared with control samples after AGR2 knockdown (Figure 3c). In T47 D cells, JC-1 staining indicated a hyperpolarization of the mitochondrial membrane after knocking down AGR2 (Figure 3d), which is required for distinct stages of both apoptosis and necrosis [31]. In apoptosis, hyperpolarization occurs early and is followed by depolarization, which was not observed at later times in siAGR2-treated T47 D cells (Supplementary figure S2b in Additional file 2), whereas sustained hyperpolarization has been reported to sensitize cells to necrosis [32]. Furthermore, consistent with necrotic, nonapoptotic cell death in T47 D cells, no increase was found in the F7-26-positive population after AGR2 knockdown (Supplementary figure S2a in Additional file 2). Based on BrdU incorporation and death assays, AGR2 knockdown in ER-positive, but not ER-negative, cells reduces cell proliferation and induces cell context-dependent death.

### AGR2 modulates critical regulators of cell growth and survival

To dissect the signaling associated with the cell-death and cell-cycle phenotypes observed in Figures 2 and 3, the impact of AGR2 knockdown on critical regulators of these pathways was assayed with Western blot. In T47 D and ZR-75-1 cells, the protein c-*Myc *and the antiapoptotic protein survivin were reduced after AGR2 knockdown (Figure 4a), consistent with the cell-death effects observed in Figure 3. To a lesser degree, survivin was also downregulated in SK-BR-3 cells treated with siAGR2. Because knockdown of AGR2 in ER-positive cells yielded an accumulation of cells in G_0_/G_1_, key regulators of this phase of the cell cycle were assayed with Western blot in these cell types. Interestingly, in all four cell types tested, cyclin D1 protein was downregulated with AGR2 silencing (Figure 4a). In addition, E2F1 was downregulated in T47 D, ZR-75-1, and MDA-MB-231 and was also consistently downregulated to a modest degree in SK-BR-3 cells (n = 3). Given that AGR2 is secreted (Figure 1), we asked whether it could act extracellularly. When ZR-75-1 cells were treated with recombinant AGR2 (Figure 4b-d), cyclin D1 was induced, beyond already significant levels, confirming that cyclin D1 is downstream of AGR2.

#### Evidence of AGR2-ER crosstalk

Previous studies showed that E2 treatment in MCF-7 cells induces AGR2 at the message level [11]. Consistent with AGR2 responsiveness to E2, E2 treatment in ZR-75-1 cells further induced AGR2 at the protein level (Figure 5a). We subsequently investigated potential crosstalk between AGR2 and ER because E2 signaling affects the cell cycle, c-*Myc*, and cyclin D1 [33], which are also modulated with AGR2 knockdown. Notably, numerous reports have been made of ER crosstalk with other pathways [34-36]. To determine whether AGR2 could conversely affect E2 signaling, ER levels were assessed after AGR2 knockdown. In ER-positive cells, silencing of AGR2 yielded a reduction of ER protein (Figure 5b). In T47 D cells, a double band for ER was detected, which may be due to a phosphorylated form of the protein. Collectively, these data suggest that E2 can affect AGR2 protein levels, and, conversely, knockdown of AGR2 can lead to a reduction in ER protein levels.

#### AGR2 biology involves ER-dependent and ER-independent mechanisms

Much of the AGR2 biology shown thus far might be tied to an impact on ER and subsequent downstream signaling. The partial antiestrogen, tamoxifen, and 'pure' antiestrogen, fulvestrant--first-line treatments for ER-positive patients--both modulate the cell cycle and reduce cyclin D1 and c-*Myc *levels [33]. In addition, in a fashion similar to AGR2 knockdown, ER is downregulated by fulvestrant. Therefore, we wanted to determine how AGR2 knockdown might affect antiestrogen activity. Phenotypically, AGR2 knockdown augmented the degree of growth inhibition when combined with either antiestrogen (Figure 6a). At the level of target modulation, silencing AGR2 in combination with antiestrogens further enhanced ER and cyclin D1 downregulation (Figure 6b, compare lane 6 with lanes 9 and 12). This suggests that an anti-AGR2 therapy would not preclude the activity of an antiestrogen, and the combination might be advantageous.

Tamoxifen treatment in MCF-7 cells has been reported to elevate Src phosphorylation levels and is associated with acquired tamoxifen resistance [37]. Recently, targeting both Src and ER has been shown to prevent acquired resistance to tamoxifen therapy [38]. To determine whether inhibiting AGR2 activity might affect this resistance-associated protein, phosphorylation of Src was examined. Silencing AGR2 downregulated p-Src, but not total Src levels, in both ER-positive breast cancer cell lines (Figure 6c).

Because cyclin D1 is a downstream target of E2 signaling, we sought to determine whether cyclin D1 modulation by AGR2 could be tied exclusively to an impact on ER. The kinetics of AGR2 knockdown was assessed at 24, 32, and 48 hours. Cyclin D1 was downregulated after 24 hours, whereas ER was downregulated at 32 hours (Figure 6d), indicating that cyclin D1 downregulation was at least, in part, due to an ER-independent mechanism. This is also consistent with the cyclin D1 downregulation observed in ER-negative cell lines (Figure 4a).

#### Anti-AGR2 Ab modulates cyclin D1 and inhibits cell growth

**Figure 7:**
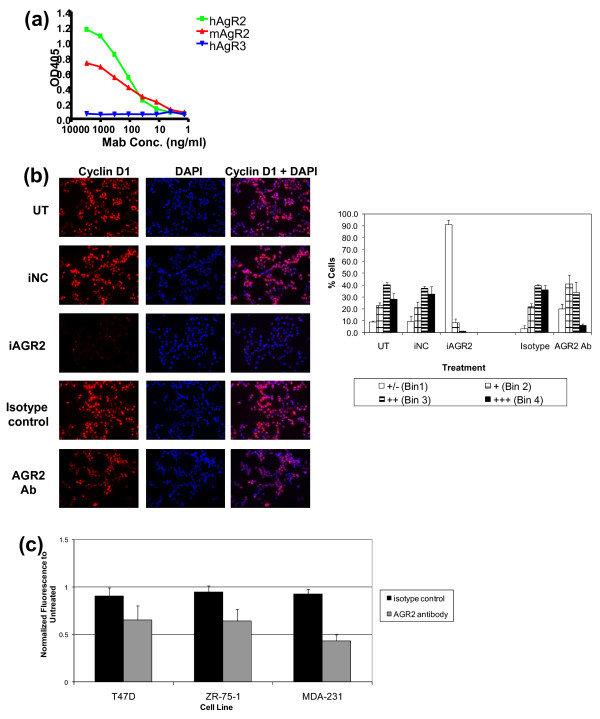
**Impact of rat anti-AGR2 Ab on cell growth and cyclin D1 in T47 D cells**. **(a) **Rat anti-AGR2 Abs were tested for AGR2 specificity by using an ELISA directed against human AGR2 and human AGR3. Species crossreactivity also was assessed by using an ELISA directed against mouse AGR2. **(b) **After confirming Ab specificity, T47 D cells were treated with an anti-AGR2 Ab (10 μg/mL) for 48 hours or AGR2 siRNA for 72 hours, and cyclin D1 modulation was examined with immunofluorescence. Cells were stained with cyclin D1, and mounting media containing DAPI were used. Images were taken by using a fluorescence microscope and pseudo-colored in Adobe Photoshop. The isotype control Ab used for cyclin D1 staining was an anti-AGR2 Ab of the same isotype but was not shown to modulate cyclin D1 or to have an impact on growth. Cyclin D1 intensity was quantitated by using ImagePro and binned based on intensity, and the percentage of cells in each bin based on cyclin D1 intensity is represented (Bin 1, weakest staining; Bin 4, brightest staining). **(c) **T47 D, ZR-75-1, and MDA-MB-231 cells were treated for 5 days with 20 μg/mL anti-AGR2 Ab. The relative number of cells was quantitated by using the MTT assay. Results are expressed relative to untreated sample for each cell line.

Although the induction of cyclin D1 protein with recombinant AGR2 suggested that AGR2 can act extracellularly (Figure 4c), given that Park *et al. *[39] had shown that AGR2 is an endoplasmic reticulum protein, we asked whether AGR2 activity could be inhibited extracellularly. Monoclonal antibodies raised in rats immunized with recombinant AGR2 were assayed for AGR2 specificity and species cross-reactivity with ELISA. The antibody bound both mouse and human forms of AGR2, but not human AGR3 (Figure 7a). Cultured T47 D cells were treated with the monoclonal Ab and compared with AGR2 siRNA treatment for effects on cyclin D1, by using cyclin D1 immunofluorescence staining (Figure 7b, left panel). Images were quantitated for relative cyclin D1 intensity (Figure 7b, right panel). Consistent with Western data (Figure 4a), cyclin D1 was reduced after transiently silencing AGR2 by siRNA in T47 D cells. Cyclin D1 was also significantly reduced in T47 D cells treated with an anti-AGR2 antibody. The impact of the AGR2 Ab on cell growth was investigated in T47 D, ZR-75-1, and MDA-MB-231 cells. Treatment with the anti-AGR2 Ab modestly reduced cell growth in all three cell lines (Figure 7c). In summary, the effects of the anti-AGR2 Ab suggest that AGR2 can act extracellularly.

## Discussion

AGR2 is overexpressed in a number of epithelial cancers [11,15-25] and is inversely associated with patient survival in the ER-positive patient population [21,25]. Although this association itself does not indicate a critical role for AGR2 in disease progression, it does warrant examination of the functional relevance of this gene. Both anchorage-dependent growth and anchorage-independent growth were inhibited after silencing endogenously expressed AGR2 in ER-positive cell lines (Figure 1). This impact on growth was seen with multiple AGR2 siRNA sequences (Supplementary figure S1 in Additional file 1), which supports the specificity of the effect of AGR2 knockdown on the inhibition of cancer cell growth. AGR2 knockdown significantly reduced colony formation in soft agar in ER-negative lines, as well (Figure 1c), raising an interesting discrepancy between ER-positive and ER-negative lines. The soft-agar assay represents a particularly stressful *in vitro *assay, as it requires the cancer cells to grow from a single cell, whereas other assays allow for cell-cell interaction. Support exists in the literature for a role of AGR2 in stress conditions [15,26]. Another possibility is that the soft-agar assay measures AGR2-dependent biologies that are not captured in the other assays, a concept consistent with distinct breast cancer models, in which AGR2 overexpression was reported to play a role in metastases and adhesion, but not growth [11]. Aligned with the hypothesis that MDA-MB-231 cells may be more sensitive to neutralization of AGR2 activity under stress conditions, we observed that our AGR2-neutralizing mAb inhibited MDA-MB-231 cell growth under low serum conditions. As noted earlier, siRNA-mediated knockdown of AGR2 in MDA-MB-231 cells in full-serum conditions had little growth effect in anchorage-dependent conditions. The MDA-MB-231 cells were more sensitive to growth inhibition with the AGR2-neutralizing mAb than were T47 D and ZR-75-1 cells, although this may be trivially explained by the observation that MDA-MB-231 cells have significantly lower levels of AGR2 than do T47 D and ZR-75-1 cells used in the same experiment and thus may be more readily neutralized. Although this is the first time a role for AGR2 has been demonstrated in breast cancer cell growth, these results are consistent with the impact of AGR2 knockdown on cell growth reported in other non-breast cancer models.

To further evaluate the role of AGR2 in breast cancer, the impact on cell death and the cell cycle were explored. Supporting the anchorage-dependent functional data, a cell-cycle phenotype and induction of cell death was seen in both T47 D and ZR-75-1 cells (Figures 2 and 3). Thus far, neither phenotype has been reported. The BrdU phenotype is in contrast to previous reports in which stable expression of AGR2 in H1299 cells had no effect on the cell cycle or DNA synthesis [15]. Cell context-dependent differences exist in the mechanisms of cell death, which may be related to the genetic background of the respective cell lines, such as the differences in p53 status, but cell death was observed in either case. That inhibition of AGR2 activity can induce cytotoxicity, in addition to being cytostatic, is particularly important for the potential to treat slow-growing tumors.

To provide support for the phenotypic effects shown with AGR2 knockdown and to understand AGR2 biology in greater depth, we investigated intracellular signaling downstream of AGR2. Mechanistic hints from the literature have been limited to a role for AGR2 in the wild-type p53 transcriptional response [15]. Because T47 D cells have mutated p53, and ZR-75-1 cells have wild-type p53, and a phenotype with AGR2 knockdown was observed in both, AGR2 biology cannot be limited to the p53 pathway. Cell-cycle modulation and induction of cell death after transient knockdown of AGR2 directed us to explore other signaling pathways that may be relevant to AGR2 biology. Modulation of cyclin D1, c-*Myc*, and E2F1 by AGR2 knockdown is consistent with the cell-cycle and anchorage-dependent growth phenotype seen in the ER-positive cell lines. The impact of AGR2 knockdown on cyclin D1 in ER-negative cell lines that did not translate into a cell-cycle phenotype was an intriguing result. Several possibilities could account for this observation. It could also be a threshold issue, requiring that a certain percentage of cyclin D1 be downregulated to result in a phenotype similar to that observed in ER-positive cells. It is of note that cyclin D1 can be downregulated through both ER-dependent and ER-independent pathways. In addition, ZR-75-1 cells have cyclin D1 amplified and are known to be driven by cyclin D1 [40], which may not be so important a driver in the ER-negative cell types tested. Collectively, downregulation of cyclin D1 in all four cell types after AGR2 knockdown or treatment with an anti-AGR2 Ab supports cyclin D1 being downstream of AGR2.

We also provide evidence that AGR2 can act extracellularly. This finding is in contrast to a recent report suggesting that AGR2, a member of the protein disulfide isomerase family, is localized in the endoplasmic reticulum and plays an essential role for mucus production [39]. Because cyclin D1 is induced with an exogenous source of AGR2 (Figure 4) and reduced with an anti-AGR2 Ab (Figure 7), and AGR2 is detected in the supernatant of breast cancer cell lines, it suggests that AGR2 may have an extracellular mechanism of action. Consistent with our data, other disulfide isomerases have also been associated with cancer-relevant biologies, including invasiveness and stress survival [41,42].

To provide further evidence that AGR2 is important in breast cancer progression, silencing AGR2 in ER-positive cells downregulated c-*Myc*, p-Src, and survivin. All of these molecules play critical roles in breast cancer progression by affecting growth, survival, angiogenesis, migration, and invasion [43], and hence are individually being investigated as targeted monotherapies [44,45]. Given the high level of biologic relevance of p-Src, survivin, and c-Myc in cancer, modulation of these key players after silencing AGR2 suggests that AGR2 may be the key in other cancers, beyond breast cancer.

E2 is another key driver and potent mitogen of breast cancer. Several articles in the literature indicate AGR2 is an E2-responsive gene [11-13], and we similarly observed an induction of AGR2 in E2-treated ZR-75-1 cells (Figure 5a). The more-novel finding is that ER protein levels are reduced after AGR2 knockdown (Figure 5b). Because ER downregulation leads to reduced estrogen responsiveness with fulvestrant, this suggests that ER downregulation induced by AGR2 knockdown might also negatively influence the mitogenic activity of E2. The relative E2 responsiveness could not be accurately assessed in these AGR2-silenced cells, given the complexity of the relations of these molecules, because E2 itself modulates ER in T47 D cells [46].

Given the similar profiles of AGR2 knockdown with E2 signaling, important considerations existed when assessing the potential crosstalk between the ER and AGR2 pathways. Initially, we asked how AGR2 knockdown would affect the effect of antiestrogens on cancer cell lines. Next, we determined whether AGR2 might have ER-independent activities. AGR2 knockdown in combination with antiestrogens did not preclude antiestrogen efficacy, and the combination enhanced the impact on growth, ER and cyclin D1 (Figure 6a, b). The kinetics of AGR2 knockdown showed that cyclin D1 downregulation occurs before ER downregulation, and therefore, AGR2 has an ER-independent pathway for downregulating cyclin D1, which is supported by the impact on cyclin D1 seen in ER-negative cells.

## Conclusions

AGR2 is commonly overexpressed in cancers, and our data suggest an important functional role for AGR2 in breast cancer. AGR2 affects key breast cancer drivers, including cyclin D1, c-*Myc*, and ER, as well as more general oncogenic signaling nodes such as p-Src and survivin. Removal of AGR2 has an impact on cancer-relevant pathways, including the cell cycle and E2 signaling, ultimately resulting in cell death, thus demonstrating that AGR2 plays a critical role in breast cancer progression. Beyond elucidating novel biology, the mechanism of action also suggests that AGR2 would be a good therapeutic target because its inhibition appears to have added benefit when combined with conventional antiestrogen treatments. Tumors treated with tamoxifen can become hypersensitive to E2 [47], and both an anti-AGR2 therapy and fulvestrant address this issue because ER is downregulated with these treatments. In addition, survivin and p-Src have been implicated in tamoxifen resistance, and both are modulated by AGR2 knockdown. Furthermore, an anti-AGR2 therapy, unlike fulvestrant, can potentially function in cancers driven by cyclin D1 that can no longer respond to E2, given the ER-independent actions of AGR2 on cyclin D1. Collectively, the data presented support the utility of an anti-AGR2 therapy in ER-positive breast cancers.

## Abbreviations

Ab: antibody; AGR2: anterior gradient-2; E2: 17-β estradiol; ER: estrogen receptor-α; IP: immunoprecipitation; rh: recombinant human.

## Competing interests

The authors declare that Schering-Plough has a patent that includes data from this article.

## Authors' contributions

KV drafted manuscript and performed cell-cycle, cell death, rhAGR2 stimulation, and ER-AGR2 crosstalk experiments. SH performed numerous functional experiments and observed cyclin D1, p-Src proteins modulated after knocking down AGR2 in breast cancer cells. LB generated anti-AGR2 Ab and performed ELISAs. JG profiled cell lines for AGR2 mRNA expression, which led to the selection of cell lines for the experiments carried out. LF expressed and purified AGR2 protein used in rhAGR2 stimulation experiments. MS carried out Taqman analysis of a panel of genes in AGR2-silenced samples, which led to the observation of a downregulation of cyclin D1. MJJ initiated and oversaw the AGR2 project, devised key experiments, and edited the manuscript.

## Supplementary Material

Additional file 1 **Supplementary figure S1**. Impact of AGR2 on breast cancer cell growth using AGR2 siRNA from multiple vendors. **(a) **T47 D and MDA-MB-231 cells were treated with AGR2 siRNA from Invitrogen, Ambion and Dharmacon and their appropriate nontargeting controls. Ninety-six hours after transfection, Cell Titer Glo was used as a readout for relative cell number. Results are expressed relative to untransfected cells. **(b) **Whole-cell lysates were isolated from T47 D and MDA-MB-231 cells at 48, 72, or 96 hours after transfection to confirm knockdown or AGR2 protein. Click here for file

Additional file 2 **Supplementary figure S2**. Additional death assays after AGR2 knockdown in T47 D cells. **(a) **F7-26 staining, a measure of ssDNA breaks, was measured 96 hours after AGR2 knockdown in T47 D cells with fluorescence-activated cell sorting (FACS) analysis. Hydrogen peroxide was used as a positive control for the assay. **(b) **Alterations in mitochondrial membrane potential 120 hours after AGR2 knockdown were assessed by determining the ratio of JC-1_red _to JC-1_green _and are represented as a ratio of the untransfected control (±SD), n = 3. MG132 and CCCP served as apoptosis and depolarization controls, respectively. Click here for file
