# Supplementary figures and images for: Anterior gradient-2 plays a critical role in breast cancer cell growth and survival by modulating cyclin D1, estrogen receptor-α and survivin
Source: Breast Cancer Res. 2010 Jun 4;12(3):R32. doi: 10.1186/bcr2586 (PMC2917027; doi:10.1186/bcr2586)

## Slide 1
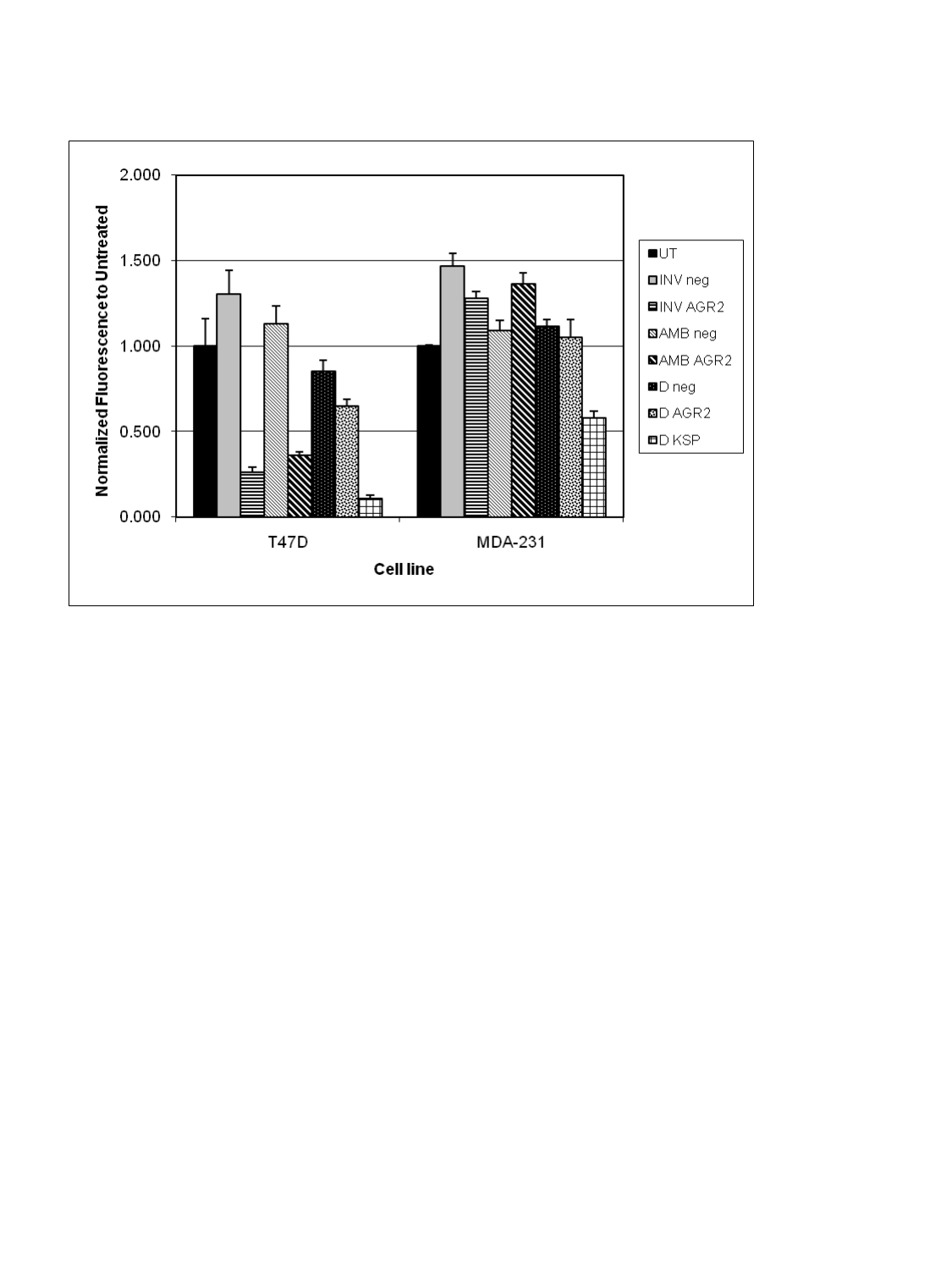

Supplement: Additional file 1 — Supplementary figure S1. Impact of AGR2 on breast cancer cell growth using AGR2 siRNA from multiple vendors. (a) T47 D and MDA-MB-231 cells were treated with AGR2 siRNA from Invitrogen, Ambion and Dharmacon and their appropriate nontargeting controls. Ninety-six hours after transfection, Cell Titer Glo was used as a readout for relative cell number. Results are expressed relative to untransfected cells. (b) Whole-cell lysates were isolated from T47 D and MDA-MB-231 cells at 48, 72, or 96 hours after transfection to confirm knockdown or AGR2 protein. [file bcr2586-S1.PPT]

## Slide 1
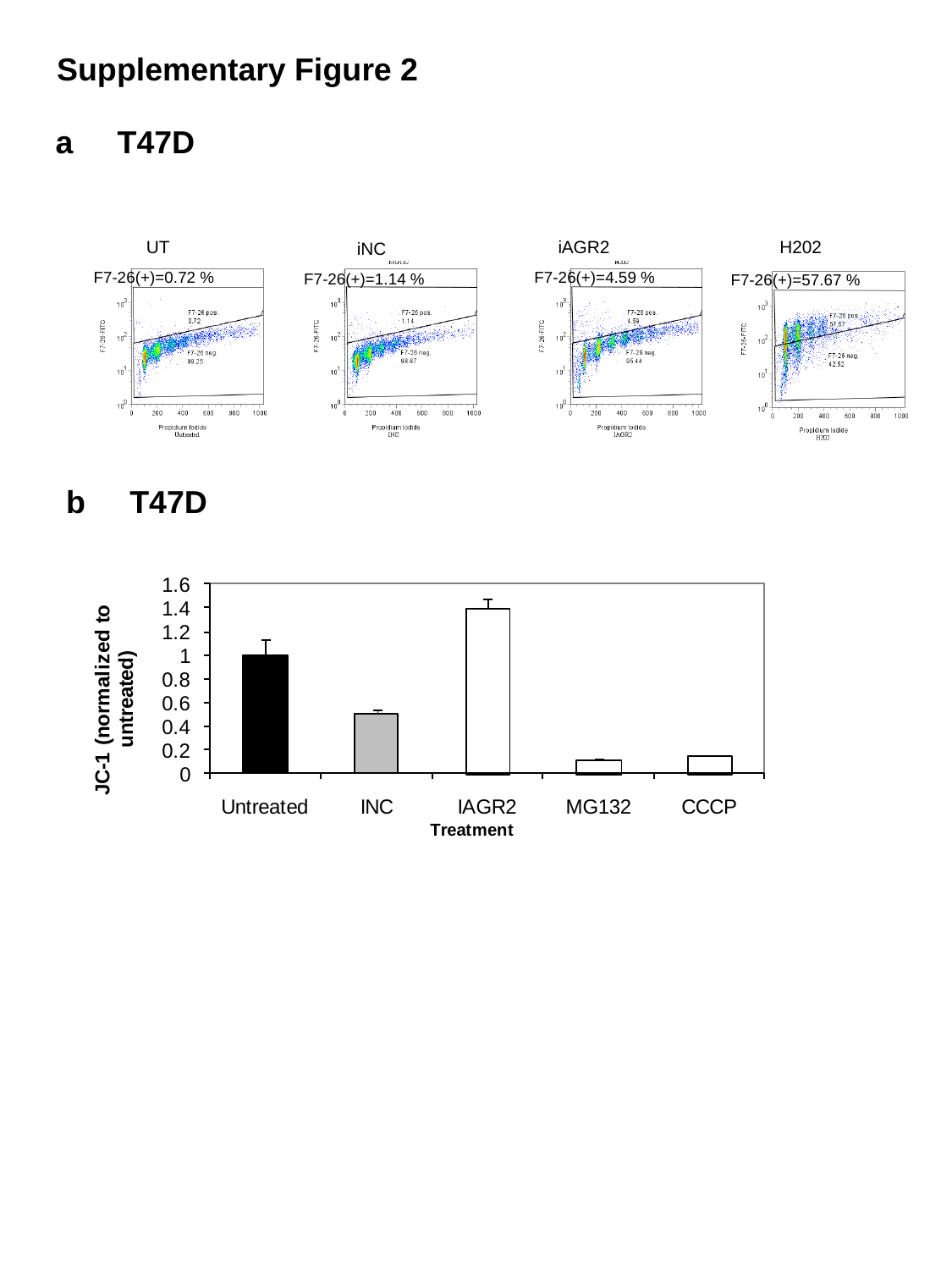

Supplementary Figure 2
a T47D
iAGR2
H202
UT
iNC
F7-26(+)=0.72 %
F7-26(+)=4.59 %
F7-26(+)=1.14 %
F7-26(+)=57.67 %
b T47D

Supplement: Additional file 2 — Supplementary figure S2. Additional death assays after AGR2 knockdown in T47 D cells. (a) F7-26 staining, a measure of ssDNA breaks, was measured 96 hours after AGR2 knockdown in T47 D cells with fluorescence-activated cell sorting (FACS) analysis. Hydrogen peroxide was used as a positive control for the assay. (b) Alterations in mitochondrial membrane potential 120 hours after AGR2 knockdown were assessed by determining the ratio of JC-1red to JC-1green and are represented as a ratio of the untransfected control (±SD), n = 3. MG132 and CCCP served as apoptosis and depolarization controls, respectively. [file bcr2586-S2.PPT]
